# Consulting to nephrologist when starting continuous renal replacement therapy for acute kidney injury is associated with a survival benefit

**DOI:** 10.1371/journal.pone.0281831

**Published:** 2023-02-15

**Authors:** Jinwoo Lee, Seong Geun Kim, Donghwan Yun, Min Woo Kang, Yong Chul Kim, Dong Ki Kim, Kook-Hwan Oh, Kwon Wook Joo, Yon Su Kim, Seung Seok Han

**Affiliations:** Department of Internal Medicine, Seoul National University College of Medicine, Seoul, Korea; University of Bari: Universita degli Studi di Bari Aldo Moro, ITALY

## Abstract

**Background:**

Several studies suggest improved outcomes for patients with kidney disease who consult a nephrologist. However, it remains undetermined whether a consultation with a nephrologist is related to a survival benefit after starting continuous renal replacement therapy (CRRT) due to acute kidney injury (AKI).

**Methods:**

Data from 2,397 patients who started CRRT due to severe AKI at Seoul National University Hospital, Korea between 2010 and 2020 were retrospectively collected. The patients were divided into two groups according to whether they underwent a nephrology consultation regarding the initiation and maintenance of CRRT. The Cox proportional hazards model was used to calculate the hazard ratio (HR) of mortality during admission to the intensive care unit after adjusting for multiple variables.

**Results:**

A total of 2,153 patients (89.8%) were referred to nephrologists when starting CRRT. The patients who underwent a nephrology consultation had a lower mortality rate than those who did not have a consultation (HR = 0.47 [0.40–0.56]; P < 0.001). Subsequently, patients who had nephrology consultations were divided into two groups (i.e., early and late) according to the timing of the consultation. Both patients with early and late consultation had lower mortality rates than patients without consultations, with HRs of 0.45 (0.37–0.54) and 0.51 (0.42–0.61), respectively.

**Conclusions:**

Consultation with a nephrologist may contribute to a survival benefit after starting CRRT for AKI.

## Introduction

Acute kidney injury (AKI) is a risk factor for mortality in critically ill patients admitted to the intensive care unit (ICU) [1–5]. Continuous renal replacement therapy (CRRT) is a treatment option for patients displaying both hemodynamic instability and severe AKI. Despite advances in CRRT techniques over the past decades, the mortality rate for patients on CRRT due to AKI remains high [4–9]. Although guidelines regarding CRRT implementation exist [10–12], complications may occur during CRRT, such as hypotension, major and minor electrolyte imbalance, hypothermia, hematological abnormalities, and catheter-related complications [13]. Accordingly, starting CRRT does not always guarantee a survival benefit, which emphasizes the importance of an individualized approach [14–17].

For patients with chronic kidney disease, early consultation with nephrologists may delay the progression of kidney dysfunction and confer a survival benefit [12, 14, 15, 18–20]. Similarly, a nephrology consultation when patients are at risk of severe AKI may be associated with improved survival outcomes [16, 17, 21–24]. Consequently, it is plausible that a multidisciplinary task force with nephrologists should also be applied to improve the outcomes of ICU patients, particularly when they have AKI or need dialysis. To date, there have been no studies on whether nephrology consultations are associated with the outcomes of severe AKI requiring CRRT. Herein, we addressed this issue using a cohort of patients undergoing CRRT after stratification by consultation time and a propensity score matching.

## Methods

### Patients and data collection

This study design was approved by the institutional review board of Seoul National University Hospital (No. H-2110-085-1262) and complied with the Declaration of Helsinki. Informed consent was waived under the approval. A total of 2,512 patients undergoing CRRT due to AKI at Seoul National University Hospital from June 2010 to December 2020 were retrospectively reviewed. Patients who were aged < 18 years (n = 24) and who had end-stage kidney disease at the time of initiating CRRT (n = 91) were excluded. Accordingly, 2,397 patients were included in the final analysis.

Baseline data, such as age, sex, weight, cause of AKI (e.g., septic or nonseptic), ICU division, use of inotropes, use of mechanical ventilation, type of central catheter, and CRRT settings (e.g., blood flow rate, target dose, and ultrafiltration), were collected. The comorbidities and severity of illness were evaluated using the Charlson comorbidity index (CCI) [25], the sequential organ failure assessment (SOFA) [26], and the acute physiology assessment and chronic health evaluation (APACHE) II [27]. The consultation with nephrologists (e.g., with DKK or SSH) and its timing were retrieved for outcome analyses.

### Nephrology consultation in CRRT patients

Consultation to nephrologist was recommended, but not mandatory. Nephrologists tried to identify the cause of AKI, and discussed the treatment options that would help renal recovery. Other discussion points included the need of CRRT initiation, optimal target dose, blood flow rate, target ultrafiltration, type of dialysates, vascular access, anticoagulants, replacement cycle of filters, and correction of electrolytes. Their roles did not differ between the timing of consultation. The implementation of CRRT due to severe AKI was adherent to the Kidney Disease Improving Global Outcomes (KDIGO) guideline [10]. To deliver 20–25 ml/kg/hr, we conducted CRRT with a target dose of 30–35 ml/kg/hr in accordance with the KDIGO guideline [10], and for some ARDS patients, the target dose was raised up to 40ml/kg/hr. In the case of a nephrology consultation where CRRT has already started, we mainly answered not only the items mentioned above, but also the timing of renal replacement therapy weaning and hemodialysis transition, etc.

### Outcomes

The primary outcome was all-cause mortality after starting CRRT. Additional outcomes, such as CRRT mortality and in-hospital mortality, were evaluated.

### Statistical analysis

Categorical and continuous variables were expressed as proportions and means ± standard deviations when they were normally distributed and as medians with interquartile ranges when they were not normally distributed. The normality of the distribution was analyzed using the Kolmogorov–Smirnov test. Either the chi-square test or the Fisher’s exact test was used to compare categorical variables. The Student’s t test and the Mann–Whitney U test were used for continuous variables with or without a normal distribution, respectively. Kaplan–Meier survival curves were drawn to determine the difference between the consultation and no consultation groups, and the significance was estimated using the log-rank test. Cox proportional hazards models with and without stepwise adjustment of multiple variables were used to calculate the hazard ratio (HR) of mortality outcomes. We tested the proportional hazard assumption using the Schoenfeld test. Because many baseline parameters differed between the consultation and no consultation groups, propensity score matching with inverse probability treatment weighting (IPTW) was additionally performed. Baseline variables, such as age, sex, weight, cause of AKI, ICU division, use of inotropes, use of mechanical ventilation, the type of central catheter, and CCI, SOFA, and APACHE II scores, were used to calculate propensity scores, but the CRRT setting variable was excluded from the calculation because the settings were decided by the clinicians. A two-way analysis of variance was performed to evaluate the effect modification of nephrologist consultation on mortality outcome in each subgroup. All statistical analyses were performed using R software (version 4.1.2; R core team, Vienna, Austria). A two-tailed P value less than 0.05 was considered statistically significant.

## Results

### Baseline characteristics

**Fig 1:**
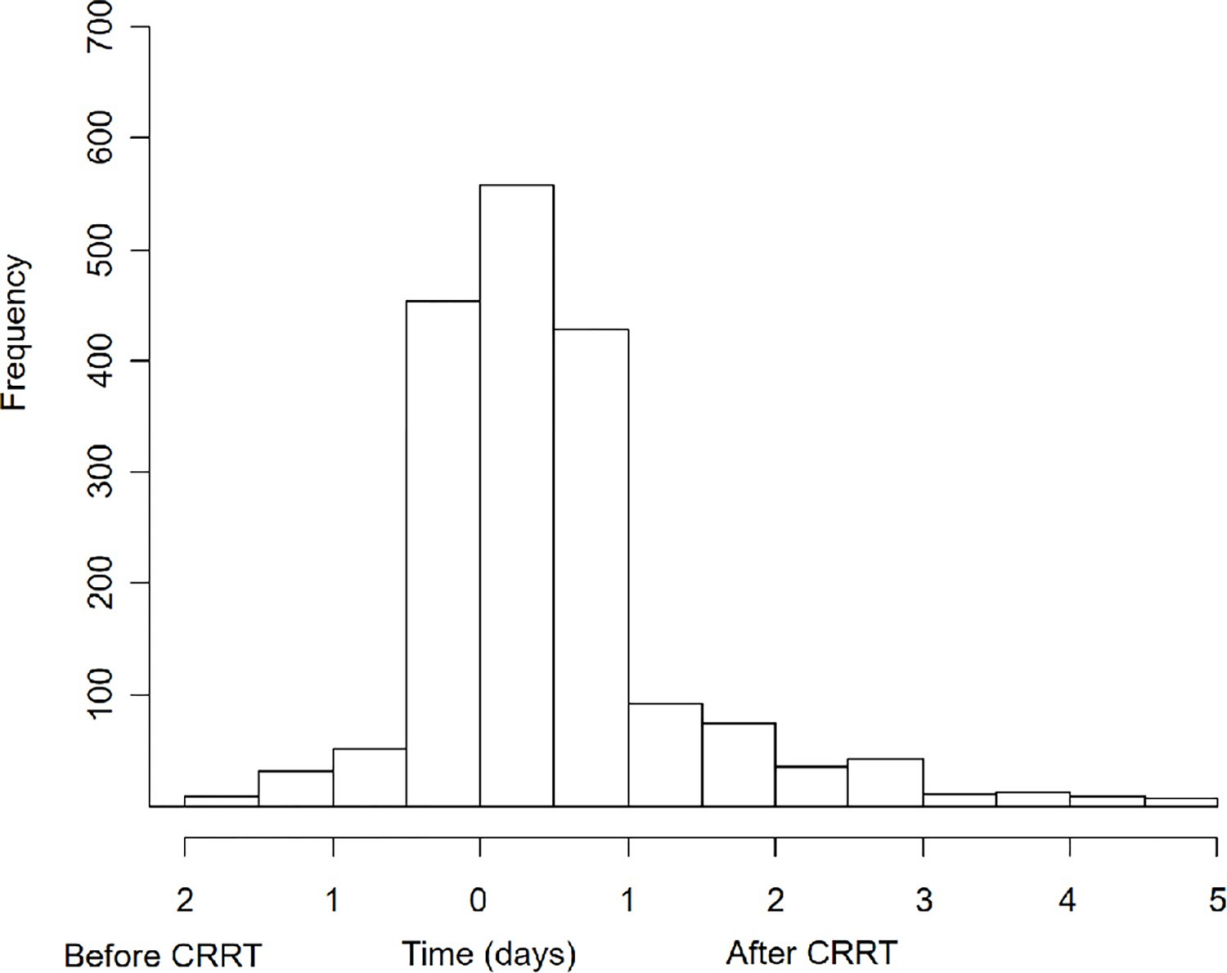
Histogram of patients with nephrology consultation according to consultation time. CRRT, continuous renal replacement therapy.

The baseline characteristics are shown in Table 1. The mean age was 64 ± 15 years, and 61.5% of the patients were female. The proportion of patients with septic AKI was 47.3%, and approximately half of the patients were hospitalized in the medical ICU. Approximately 50% and 80% of patients used inotropes and mechanical ventilation, respectively. A total of 2,153 patients (89.8%) received guidance on CRRT initiation and maintenance from nephrologists. Fig 1 shows the distribution of the timing of nephrology consultations. The median consultation time was 10 hours (interquartile range, 16–22 hours) after CRRT initiation. Seventy percent of consultations were performed within 24 hours of CRRT initiation. 38.0% of consultations were made before starting CRRT, and other consultations were conducted after starting CRRT. SOFA and APACHE II scores were higher in the no consultation group than in the consultation group, but other variables did not differ between the two groups.

**Table 1:** Baseline patient characteristics.

Variables	Total (n = 2,397)	Consultation (n = 2,153)	No consultation (n = 244)	P
Age (year)	64.1 ± 14.9	64.1 ± 14.8	63.7 ± 15.7	0.6
Male (%)	61.5	61.4	62.3	0.8
Weight (kg)	61.8 ± 13.3	61.8 ± 13.4	61.9 ± 12.5	0.9
Septic acute kidney injury (%)	47.3	46.7	52.8	0.1
ICU division (%)				0.3
MICU	50.7	50.5	52.5	
SICU	19.1	19.1	18.9	
CPICU	12.1	12.4	9.0	
EICU	17.9	17.8	18.4	
DICU	0.2	0.1	1.2	
Inotropes (%)	50.8	50.6	52.9	0.5
Mechanical ventilation (%)	78.0	77.5	82.0	0.1
Catheter (%)				0.9
Intrajugular	37.5	37.5	37.3	
Femoral	52.2	52.3	52.0	
Subclavian	10.3	10.2	10.7	
Blood flow rate (ml/min)	110.9 ± 24.5	110.9 ± 24.2	110.8 ± 26.9	0.9
Target dose (ml/kg/hr)	42.2 ± 15.2	42.0 ± 15.1	43.8 ± 16.5	0.09
Target UF (ml/day)	0 (0–500)	200 (0–500)	0 (0–500)	0.3
CCI score	3.3 ± 2.3	3.4 ± 2.3	3.3 ± 2.4	0.6
SOFA score	11.9 ± 3.7	11.8 ± 3.7	13.3 ± 3.3	<0.001
APACHE II score	26.2 ± 7.8	25.8 ± 7.7	29.0 ± 8.0	<0.001

Abbreviations: ICU, intensive care unit; MICU, medical intensive care unit; SICU, surgical intensive care unit; CPICU, cardio-pulmonary intensive care unit; EICU, emergency intensive care unit; DICU, disaster intensive care unit for COVID-19 infection; UF, ultrafiltration; CCI, Charlson comorbidity index; SOFA, sequential organ failure assessment; APACHE, acute physiologic and chronic health evaluation.

### Association between consultation and survival

During a median follow-up period of 10 days (interquartile range, 3–28 days), 1,592 patients (66.4%) died. The incidence of mortality was 26.7 deaths per 1,000 person-days. When a univariate Cox proportional hazards model was conducted, certain variables, such as cause of AKI, ICU division, use of mechanical ventilation, type of catheter, blood flow rate, target ultrafiltration, and CCI, SOFA and APACHE II scores, were significant factors related to all-cause mortality (Table 2), and all of these were considered adjusting variables in subsequent multivariate regression models.

**Table 2:** Risk factors related to all-cause mortality.

Variables	Unadjusted HR (95% CI)	P	Adjusted HR (95% CI)*	P
Age (per 1 year)	1.004 (1.000–1.007)	0.03	1.002 (0.998–1.006)	0.4
Female (vs. male)	0.987 (0.887–1.098)	0.8	0.963 (0.849–1.093)	0.6
Weight (per 1 kg)	0.999 (0.995–1.002)	0.5	0.989 (0.984–0.994)	<0.001
Nonseptic acute kidney injury (vs. septic)	0.670 (0.596–0.752)	<0.001	0.897 (0.792–1.017)	0.09
ICU division				
MICU	Reference		Reference	
SICU	0.556 (0.480–0.644)	<0.001	0.594 (0.501–0.704)	<0.001
CPICU	0.340 (0.277–0.418)	<0.001	0.304 (0.236–0.391)	<0.001
EICU	0.938 (0.815–1.080)	0.4	0.883 (0.746–1.046)	0.2
DICU	0.549 (0.177–1.706)	0.3	0.403 (0.129–1.261)	0.1
Inotropes (vs. none)	1.057 (0.953–1.173)	0.3	1.011 (0.898–1.137)	0.9
Mechanical ventilation (vs. none)	1.646 (1.431–1.895)	<0.001	1.196 (0.997–1.434)	0.05
Catheter				
Intrajugular	Reference		Reference	
Femoral	1.128 (1.008–1.262)	0.04	1.070 (0.937–1.221)	0.3
Subclavian	1.179 (0.986–1.410)	0.07	0.938 (0.767–1.148)	0.5
Blood flow rate (per 10 ml/min)	1.002 (1.000–1.004)	0.05	1.010 (0.983–1.038)	0.5
Target dose (per 1 ml/kg/hr)	1.003 (0.999–1.006)	0.1	0.996 (0.992–0.999)	0.04
Target ultrafiltration (per 100 ml/day)	0.985 (0.977–0.994)	<0.001	0.987 (0.976–0.998)	0.02
CCI score (per 1 unit)	1.041 (1.018–1.064)	<0.001	1.038 (1.012–1.064)	0.003
SOFA score (per 1 unit)	1.150 (1.132–1.167)	<0.001	1.107 (1.083–1.131)	<0.001
APACHE II score (per 1 unit)	1.069 (1.061–1.076)	<0.001	1.033 (1.023–1.043)	<0.001

*Adjusted for age, sex, weight, cause of acute kidney injury, ICU division, use of inotropes and mechanical ventilation, catheter type, blood flow rate, target dose, target ultrafiltration, CCI, SOFA, and APACHE II scores.

**Fig 2:**
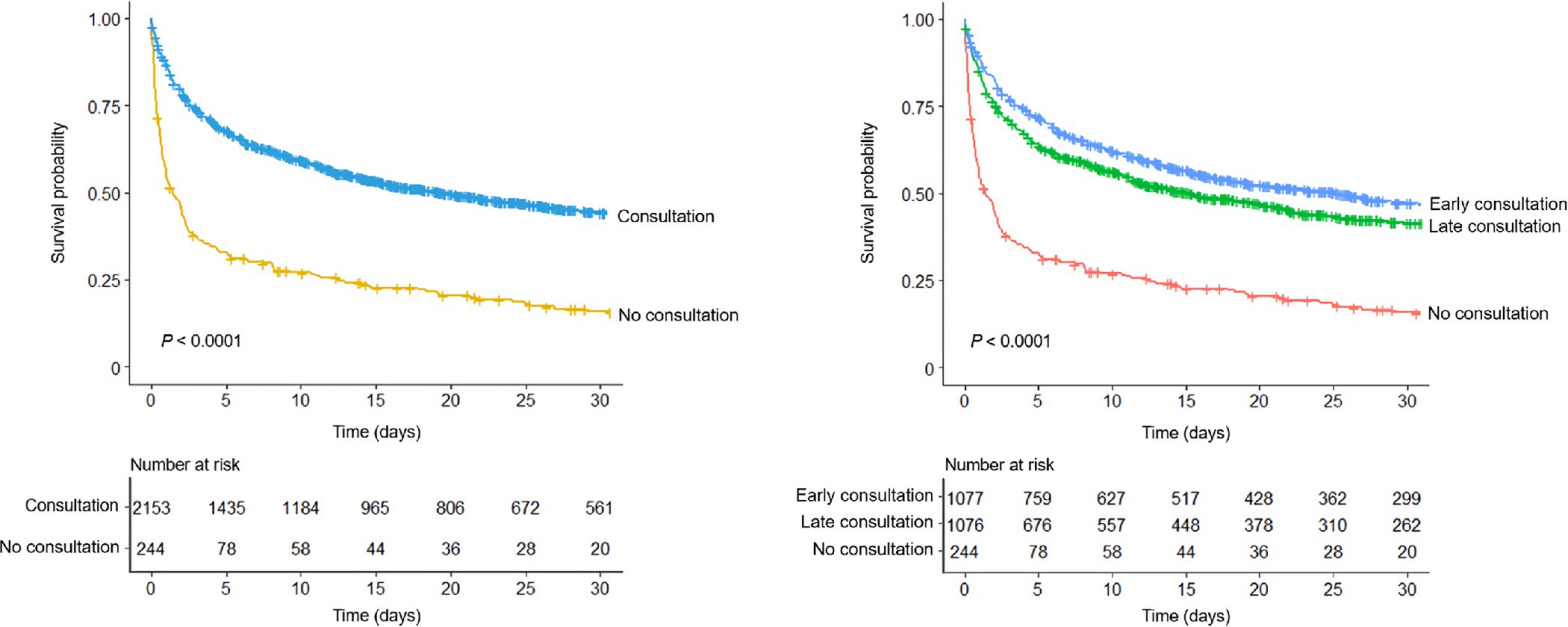
Kaplan–Meier survival curves (A) between consultation and no consultation groups or (B) among early and late consultation and no consultation groups.

**Fig 3:**
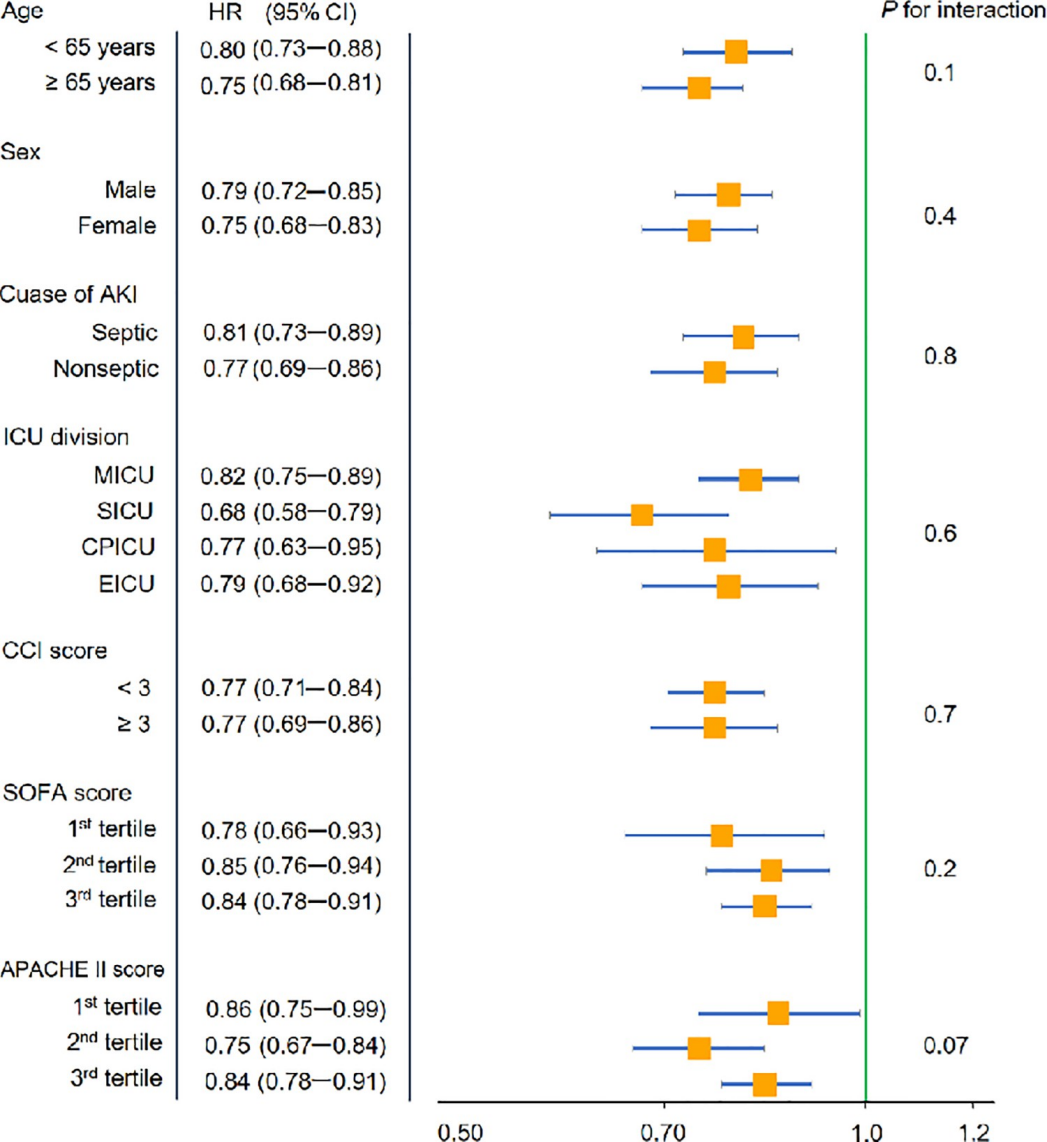
Forest plot of subgroup analysis for the hazard ratio (HR) of all-cause mortality in the consultation group compared with the no consultation group. CI, confidence interval; AKI acute kidney injury; ICU, intensive care unit; MICU, medical intensive care unit; SICU, surgical intensive care unit; CPICU, cardio-pulmonary intensive care unit; EICU, emergency intensive care unit; DICU, disaster intensive care unit for COVID-19 infection; CCI, Charlson comorbidity index; SOFA, sequential organ failure assessment; APACHE, acute physiologic and chronic health evaluation.

The all-cause mortality rates in the consultation and no consultation groups were 64.1% and 86.1%, respectively (P < 0.001). The rates of CRRT and in-hospital mortality in the consultation group were lower than those in the no consultation group (80.3% and 82.8%, respectively) (P < 0.001). Fig 2A shows the survival curves of the two groups, and the curve trends were different from each other (P < 0.001). The mortality risk was higher in the no consultation group than in the consultation group, irrespective of adjusting for multiple variables (Table 3). When all variables were adjusted, nephrologist consultation was associated with a 50% or greater reduction in mortality based on HR values. The survival benefit in the consultation group compared with the no consultation group remained consistent in the subgroup analysis (Fig 3). We further divided patients into two groups based on the median time of consultation: the early and late consultation groups. As shown in Fig 2B, the survival curves of the early and late consultation groups were separated. When multiple variables were adjusted, the absolute HR values were lower in the early consultation group than in the late consultation group (Table 4). The mortality outcomes were better in consultation groups than in no consultation group, regardless of pre- or post-CRRT consultation. (P < 0.001, S3 Table).

**Table 3:** Hazard ratio of all-cause mortality in patients with consultation compared with those without consultation.

	Hazard ratio (95% confidence interval)	P
Model 1	0.381 (0.328–0.443)	<0.001
Model 2	0.377 (0.324–0.437)	<0.001
Model 3	0.481 (0.406–0.570)	<0.001
Model 4	0.474 (0.400–0.562)	<0.001

Model 1: Unadjusted.

Model 2: Adjusted for age and sex.

Model 3: Model 2 plus weight, cause of acute kidney injury, ICU division, CCI, SOFA, and APACHE II scores.

Model 4: Model 3 plus use of inotropes and mechanical ventilation, catheter type, blood flow rate, target dose, and target ultrafiltration.

**Table 4:** Relationship between all-cause mortality and consultation time.

	Groups	Hazard ratio (95% confidence interval)	P
Model 1	No consultation	Reference	
	Late consultation	0.594 (0.547–0.644)	<0.001
	Early consultation	0.417 (0.356–0.489)	<0.001
Model 2	No consultation	Reference	
	Late consultation	0.590 (0.544–0.641)	<0.001
	Early consultation	0.411 (0.351–0.482)	<0.001
Model 3	No consultation	Reference	
	Late consultation	0.515 (0.429–0.617)	<0.001
	Early consultation	0.446 (0.371–0.537)	<0.001
Model 4	No consultation	Reference	
	Late consultation	0.506 (0.421–0.609)	<0.001
	Early consultation	0.447 (0.371–0.538)	<0.001

Model 1: Unadjusted.

Model 2: Adjusted for age and sex.

Model 3: Model 2 plus weight, cause of acute kidney injury, ICU division, CCI, SOFA, and APACHE II scores.

Model 4: Model 3 plus use of inotropes and mechanical ventilation, catheter type, blood flow rate, target dose, and target ultrafiltration.

**Fig 4:**
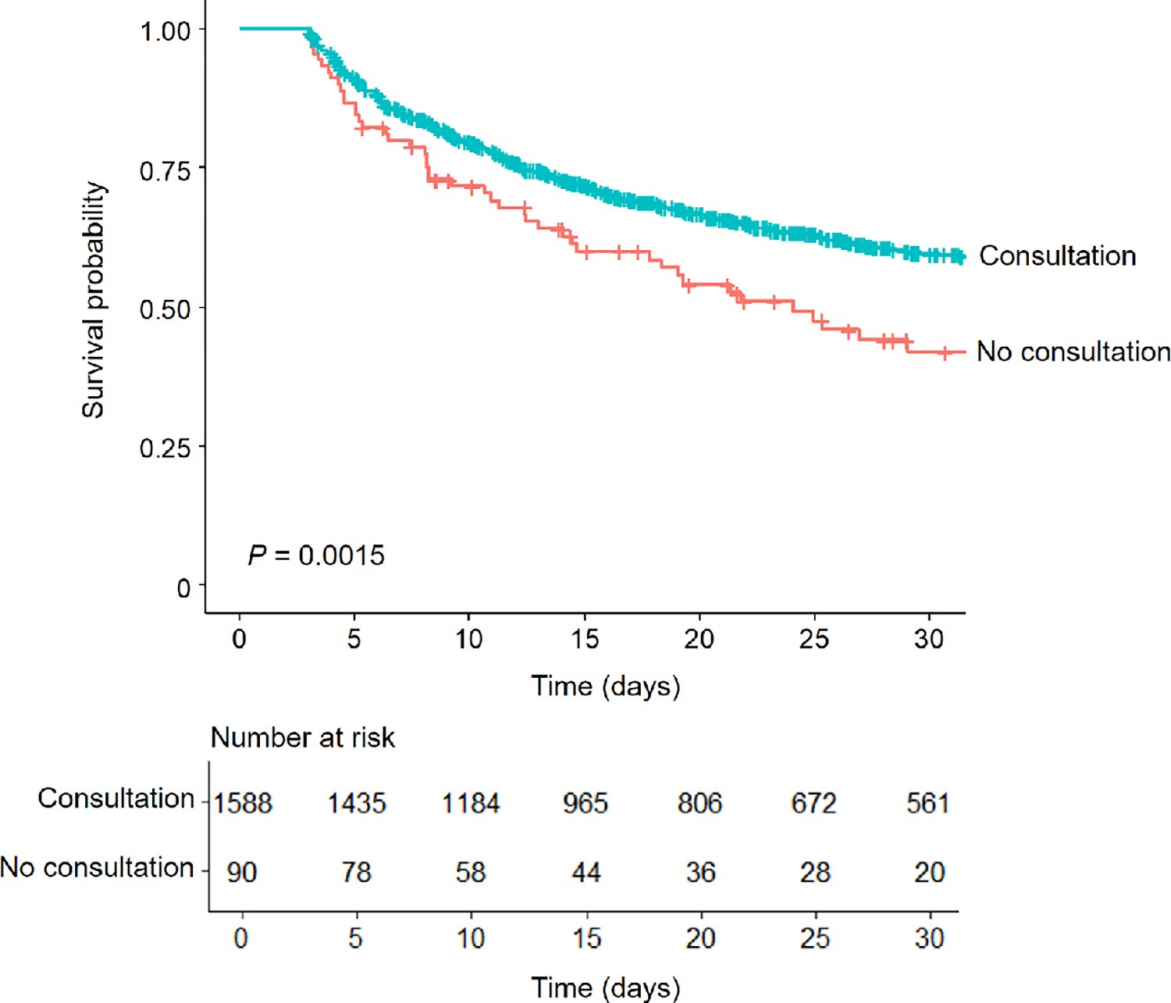
Kaplan-Meir survival curves between patients with and without consultation, excluding those who died within 72 hours after initiation of CRRT.

The survival analysis in the patients who survived after 72 hours of starting CRRT also confirmed a difference between the consultation and no consultation groups (Fig 4). Accordingly, the presence of consultation separated the survival rates even in patients who lived at the early phase of CRRT.

Because baselines differed between the consultation and no consultation groups, we matched propensity scores using two methods. S1 and S2 Tables show the similarity between the groups at baseline after matching. Despite matching propensity scores, the mortality rates in the early and late consultation groups were lower than those in the no consultation group (Table 5).

**Table 5:** Comparison of the mortality risk after propensity score matching.

Matching method	Groups	HR (95% CI)	P
IPTW-LR	No consultation	Reference	
	Late consultation	0.490 (0.393–0.612)	<0.001
	Early consultation	0.456 (0.366–0.568)	<0.001
IPTW-XGboost	No consultation	Reference	
	Late consultation	0.534 (0.393–0.726)	<0.001
	Early consultation	0.495 (0.364–0.673)	<0.001

Abbreviations: LR, logistic regression; XGboost, extreme gradient boosting.

## Discussion

A multidisciplinary approach with the help of specialists is a critical step to improve the survival outcomes of patients starting CRRT. Herein, we addressed the claim that a nephrology consultation was associated with a survival benefit in patients with AKI requiring CRRT. This trend remained consistent irrespective of several baselines, and early consultation seemed to be associated with better outcomes than late consultation.

The beneficial effect of nephrology consultations on patient outcomes has been documented in the field of nephrology, particularly when patients have chronic kidney dysfunction. Early consultation was related to the lower risks of hypoalbuminemia, anemia, disease progression, and death than late consultation [19]. This trend may be consistent even when patients receive hemodialysis, with the observation that a functioning vascular access is more prevalent in the early consultation group than in the late consultation group [18].

As is the case for patients with chronic kidney disease, a nephrology consultation may have benefits for patients during treatment for AKI. The development of an alerting system for in-hospital AKI with early consultation further notified several specialists and improved patient outcomes [16, 22]. Regarding the ICU setting, early consultation was associated with a survival benefit in patients with AKI [17, 23]. The present study first addressed the benefit of nephrology consultations in severe AKI patients requiring CRRT.

The beneficial effects of consultation on the CRRT outcome may be attributable to the following reasons. Individualized nephrology consultation on CRRT includes target dose, target ultrafiltration, blood flow rate, dialysate type, filter replacement cycles, catheter type, and anticoagulation strategy. These CRRT conditions should be taken into consideration based on several factors, such as comorbidities, cause of AKI, hemodynamic and systemic volume status, urine output, coagulation status, metabolic need, and electrolyte imbalance. The risk of CRRT complications should be closely monitored, such as intradialytic hypotension, filter clot, hypophosphatemia, hypokalemia, hypomagnesemia, and catheter thrombosis. Nephrologists may have provided an appropriate solution for these CRRT complications.

The present study had strengths such as no missing values and concrete analyses. Nevertheless, there are certain limitations to be discussed. The study design was retrospective in nature and could not determine causality between nephrology consultation and survival outcomes. In particular, since the patients who underwent CRRT in the ICU were quite complicated, all variables could not be controlled. Therefore, as previously mentioned, this retrospective study may suggest a correlation between nephrology consultation and beneficial survival outcome, but it does not imply causal inference, even though we used matching methods to overcome them. Last, because we did not consider the fluctuating trend of biochemical and setting parameters, this could affect the study results.

In conclusion, a nephrology consultation is associated with a survival benefit in patients requiring CRRT due to AKI. Future prospective randomized clinical trials are needed to determine the causality between them, and to clarify correlation with other outcomes, such as renal recovery and weaning from renal replacement therapy. The present results will be a conceptual basis for these future trials.

## Supporting information

S1 Table Baseline patient characteristics after multinomial logistic regression with inverse probability treatment weighting-based propensity score matching. (DOCX)

S2 Table Baseline patient characteristics after extreme gradient boosting model with inverse probability treatment weighting-based propensity score matching. (DOCX)

S3 Table Mortality outcomes in all patients. (DOCX)
