# Supplementary material for: Consulting to nephrologist when starting continuous renal replacement therapy for acute kidney injury is associated with a survival benefit
Source: PLoS One. 2023 Feb 15;18(2):e0281831. doi: 10.1371/journal.pone.0281831 (PMC9931119; doi:10.1371/journal.pone.0281831)
Supplement: S1 Table — (DOCX) [file pone.0281831.s001.docx]

**Supporting information**

Table S1: Baseline patient characteristics after multinomial logistic regression with inverse probability treatment weighting-based propensity score matching.

| Variables | No consultation  (n = 1,341) | Late consultation  (n = 1,680) | Early consultation  (n = 1,649) | P |
| --- | --- | --- | --- | --- |
| Age (year) | 63.1 ± 15.2 | 63.3 ± 14.5 | 64.1 ± 14.5 | 0.5 |
| Male (%) | 62.9 | 61.1 | 62.5 | 0.8 |
| Weight (kg) | 60.9 ± 12.1 | 61.6 ± 13.0 | 61.3 ± 12.8 | 0.8 |
| Septic acute kidney injury (%) | 50.9 | 51.2 | 53.4 | 0.7 |
| ICU division (%) |  |  |  | 0.1 |
| MICU | 59.5 | 54.1 | 54.4 |  |
| SICU | 14.9 | 18.4 | 19.8 |  |
| CPICU | 10.1 | 11.6 | 12.7 |  |
| EICU | 14.3 | 15.7 | 13 |  |
| DICU | 1.2 | 0.1 | 0.1 |  |
| Inotropes (%) | 55.4 | 53.6 | 52.7 | 0.7 |
| Mechanical ventilation (%) | 81.1 | 81.5 | 80.7 | 0.9 |
| Catheter (%) |  |  |  | 0.7 |
| Intrajugular | 30.0 | 33.9 | 34.3 |  |
| Femoral | 59.3 | 55.6 | 54.2 |  |
| Subclavian | 10.7 | 10.5 | 11.5 |  |
| CCI score | 2.0 ± 2.1 | 2.2 ± 2.1 | 2.3 ± 2.2 | 0.2 |
| SOFA score | 12.7 ± 3.6 | 12.2 ± 3.6 | 12.1 ± 3.7 | 0.2 |
| APACHE II score | 27.4 ± 7.7 | 26.2 ± 7.7 | 26.0 ± 7.5 | 0.1 |
