# Supplementary material for: Consulting to nephrologist when starting continuous renal replacement therapy for acute kidney injury is associated with a survival benefit
Source: PLoS One. 2023 Feb 15;18(2):e0281831. doi: 10.1371/journal.pone.0281831 (PMC9931119; doi:10.1371/journal.pone.0281831)
Supplement: S2 Table — (DOCX) [file pone.0281831.s002.docx]

**Supporting information**

Table S2: Baseline patient characteristics after extreme gradient boosting model with inverse probability treatment weighting-based propensity score matching.

| Variables | No consultation  (n = 1,308) | Late consultation  (n = 1,716) | Early consultation  (n = 1,706) | P |
| --- | --- | --- | --- | --- |
| Age (year) | 63.3 ± 15.0 | 63.6 ± 14.6 | 63.9 ± 14.7 | 0.9 |
| Male (%) | 66.0 | 61.3 | 62.2 | 0.4 |
| Weight (kg) | 62.2 ± 13.0 | 61.5 ± 13.2 | 61.4 ± 13.2 | 0.8 |
| Septic acute kidney injury (%) | 51.7 | 51.2 | 53.5 | 0.8 |
| ICU division (%) |  |  |  | 0.2 |
| MICU | 55.0 | 53.9 | 54.9 |  |
| SICU | 17.2 | 18.6 | 19.4 |  |
| CPICU | 11.6 | 11.6 | 12.6 |  |
| EICU | 13.8 | 15.8 | 13.0 |  |
| DICU | 2.4 | 0.1 | 0.1 |  |
| Inotropes (%) | 53.7 | 53.2 | 53.5 | 0.9 |
| Mechanical ventilation (%) | 80.9 | 80.9 | 80.7 | 0.9 |
| Catheter (%) |  |  |  | 0.9 |
| Intrajugular | 33.7 | 33.7 | 34.1 |  |
| Femoral | 56.3 | 56.1 | 54.5 |  |
| Subclavian | 10.0 | 10.2 | 11.4 |  |
| CCI score | 2.1 ± 2.1 | 2.3 ± 2.2 | 2.4 ± 2.3 | 0.3 |
| SOFA score | 12.5 ± 3.6 | 12.2 ± 3.7 | 12.1 ± 3.7 | 0.4 |
| APACHE II score | 26.8 ± 8.2 | 26.1 ± 7.8 | 26.1 ± 7.6 | 0.6 |
