# Supplementary material for: Consulting to nephrologist when starting continuous renal replacement therapy for acute kidney injury is associated with a survival benefit
Source: PLoS One. 2023 Feb 15;18(2):e0281831. doi: 10.1371/journal.pone.0281831 (PMC9931119; doi:10.1371/journal.pone.0281831)
Supplement: S3 Table — (DOCX) [file pone.0281831.s003.docx]

**Supporting information**

Table S3. Mortality outcomes in all patients

| Outcomes | Total  (n = 2,153) | No consultation  (n = 244) | Pre-CRRT  (n = 819) | Post-CRRT  (n = 1,334) | P |
| --- | --- | --- | --- | --- | --- |
| CRRT mortality (%) | 54.9 | 80.3 | 50.8 | 52.8 | <0.001 |
| ICU mortality (%) | 59.5 | 82.8 | 55.6 | 57.7 | <0.001 |
